# Production of β-Maltooligosaccharides of α- and δ-Tocopherols by *Klebsiella pneumoniae* and Cyclodextrin Glucanotransferase as Anti-allergic Agents

**DOI:** 10.3390/molecules14083106

**Published:** 2009-08-20

**Authors:** Kei Shimoda, Masaaki Akagi, Hiroki Hamada

**Affiliations:** 1 Department of Chemistry, Faculty of Medicine, Oita University, 1-1 Hasama-machi, Oita 879-5593, Japan; 2 Department of Pharmacology, Faculty of Pharmaceutical Sciences, Tokushima Bunri University, Yamashiro-cho, Tokushima 770-8514, Japan; 3 Department of Life Science, Okayama University of Science, 1-1 Ridai-cho, Okayama 700-0005, Japan

**Keywords:** tocopherol, *Klebsiella pneumoniae*, cyclodextrin glucanotransferase, β-maltooligosaccharide, anti-allergic activity

## Abstract

The glycosylation of α- and δ-tocopherols using *Klebsiella pneumoniae* and cyclodextrin glucanotransferase (CGTase) was investigated. *K. pneumoniae* converted α- and δ-tocopherols into the corresponding β-glucosides in 10 and 8% yield, respectively. CGTase glycosylated α-tocopheryl β-glucoside to α-tocopheryl β-maltoside (51%) and α-tocopheryl β-maltotrioside (35%). On the other hand, δ-tocopheryl β-glucoside was converted into the corresponding β-maltoside (45%) and β-maltotrioside (29%) by CGTase. The β-glucoside of α-tocopherol, and β-glucoside and β-maltoside of δ-tocopherol showed inhibitory effects on IgE antibody production and on histamine release from rat peritoneal mast cells.

## 1. Introduction

Tocopherols have been known to be essential nutrients for reproduction since 1922 [1]. They function as chain-breaking antioxidants that prevent the propagation of free radical reactions [2,3]. Recently, tocopherols have attracted much attention clinically because of their potential to be very useful drugs, and have been widely studied for their anti-aging, anticancer, anti-atherosclerosis, anticarcinogenesis, and anti-allergic effects [4,5,6,7,8,9]. However, tocopherols are lipophilic compounds that are poorly absorbed after oral administration [10] and these shortcomings prevent further pharmacological exploitation of tocopherols.

Glycosylation is a characteristic reaction which converts water-insoluble and unstable aromatic compounds into the corresponding water-soluble and stable compounds to improve their bioavailability and pharmacological properties. Therefore, tocopheryl glycosides (tocopherol derivatives) are of importance for the food and drug industries. Considerable effort has been made so far to synthesize tocopheryl glycosides by chemical methods to enhance the water-solublility of tocopherols [10,11]. Additionally, the tocopheryl glycosides have been reported to show anti-allergic activity [10,11]. We report here the glycosylation of α- and δ-tocopherols by two biocatalysts, *Klebsiella pneumoniae* and cyclodextrin glucanotransferase (CGTase), into the corresponding β-glucosides and β-malto-oligosaccharides as tocopherol derivatives which have potent anti-allergic activity. The tocopheryl glycosides synthesized here could be useful as food additives with anti-allergic activity.

## 2. Results and Discussion

### 2.1. Production of the β-glycosides of α- and δ-tocopherols

**Scheme 1:**
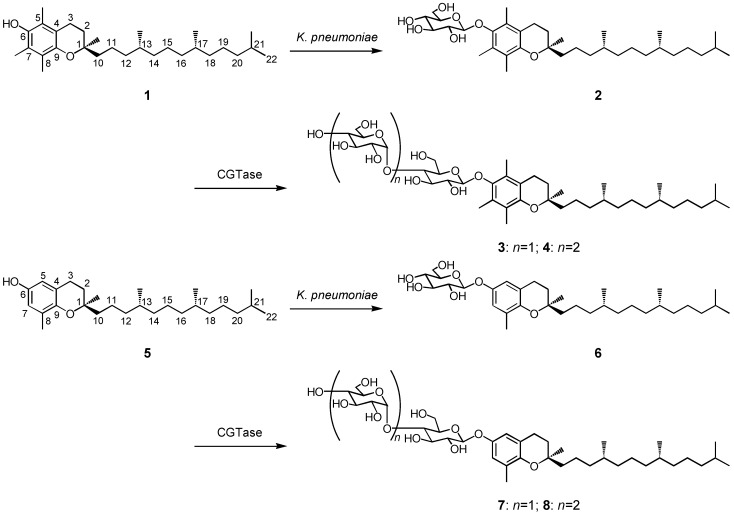
Synthetic route to α-tocopheryl β-maltooligosaccharides **3**-**4** and δ-tocopheryl β-maltooligosaccharides **7**-**8** through two-step glycosylation with *K. pneumoniae* and CGTase.

Some bacteria, *i.e., K. pneumoniae*, *Xanthomonas campestris*, and *Lactobacillus delbrueckii*, are known to produce exopolysaccharides which are excreted into the culture medium [12,13,14]. These bacteria are expected to convert exogenous lipophilic food ingredients such as tocopherols to their water-soluble glycosides. The biotransformation of α-tocopherol (**1**) was investigated using *K. pneumoniae* as a biocatalyst. After 48 h incubation of *K. pneumoniae* with α-tocopherol (**1**, 860 mg), its β-d-glucopyranoside **2** (118 mg) was obtained in 10% yield. The substrate **1** was subjected to the same biotransformation system using *X. campestris* and *L. delbrueckii*, and the compound **2** was produced in 7 (83 mg) and 5% (59 mg) yield, respectively. The β-glucoside **2** (59 mg) was subjected to further glycosylation by CGTase to give α-tocopheryl β-maltoside (**3**, 38 mg) and α-tocopheryl β-maltotrioside (**4**, 32 mg) with 51 and 35% yield, respectively. The structure of α-tocopheryl glycosides **3** and **4** was determined by comparison of their NMR data with previously reported ones [15]. The synthetic route of α-tocopheryl glycosides **2**-**4** is shown in Scheme 1. On the other hand, δ-tocopherol (**5**, 804 mg) was glucosylated to δ-tocopheryl β-d-glucopyranoside (**6**, 90 mg) in 8% yield by *K. pneumoniae*. The product **6** was obtained in 2% (23 mg) yield by glycosylation of **5** with *X. campestris*, and a trace amount of **6** was produced by *L. delbrueckii*. The *K. pneumoniae* cells were the most effective biocatalysts for the production of tocopheryl glucosides among the three bacteria. The β-glucoside **6** (56 mg) was transformed to two new compounds **7** (33 mg) and **8** (26 mg) with 45 and 29% yield, respectively, by CGTase (Scheme 1). The chemical structures of products **7** and **8** were determined as δ-tocopheryl β-maltoside and δ-tocopheryl β-maltotrioside, which have not been identified before, by HRFABMS, ^1^H- and ^13^C-NMR, H-H COSY, C-H COSY, and HMBC spectra. 

The HRFABMS spectrum of compound **7** showed a [M+Na]^+^ peak at *m*/*z* 749.4455, suggesting a molecular formula of C_3__9_H_66_O_12_ (calcd. for C_3__9_H_66_O_12_Na 749.4452 ). The ^1^H- and ^13^C-NMR data of the sugar moiety of **7** agreed with those of maltose [16]. Two proton signals at *δ* 4.41 (1H, *d*, *J* = 7.6 Hz) and 5.11 (1H, *d*, *J* = 3.2 Hz) were observed in the ^1^H-NMR spectrum of **7**, indicating that the glucoside linkage in **7** had β- and α-orientations. Each signal in the NMR spectra of **7** was assigned by H-H COSY, C-H COSY, and HMBC analyses. The HMBC spectrum of **7** showed correlations between the proton signal at *δ* 4.41 (H-1') and the carbon signal at *δ* 151.5 (C-6), and between the proton signal at *δ* 5.11 (H-1'') and the carbon signal at *δ* 80.7 (C-4'), which confirmed that the inner β-d-glucopyranosyl residue was attached to the 6-hydroxyl group of δ-tocopherol and that the second α-d-glucopyranosyl residue and the inner β-d-glucopyranosyl residue were 1,4-linked. Therefore, the structure of **7** was determined to be δ-tocopheryl β-maltoside.

Product **8** showed a pseudomolecular [M+Na]^+^ ion peak at *m*/*z* 911.4851 (HRFABMS), consistent with a molecular formula of C_45_H_76_O_1__7_ (calcd. for C_45_H_76_O_1__7_Na 911.4880 ). The ^1^H-NMR spectrum of **8** showed three proton signals at *δ* 4.37 (1H, *d*, *J* = 8.0 Hz), 4.59 (1H, *d*, *J* = 3.6 Hz), and 5.10 (1H, *d*, *J* = 3.2 Hz), which indicated the presence of one β-anomer and two α-anomers in the sugar moiety. The sugar component in **8** was shown to be maltotriose based on the coupling pattern of the sugar proton signals and the chemical shifts of the sugar carbon signals [16]. HMBC correlations between the proton signal at *δ* 4.37 (H-1') and the carbon signal at *δ* 151.5 (C-6), between the proton signal at *δ* 4.59 (H-1'') and the carbon signal at *δ* 80.5 (C-4'), and between the proton signal at *δ* 5.10 (H-1''') and the carbon signal at *δ* 81.5 (C-4'') established that the inner β-d-glucopyranosyl residue was attached to the 6-hydroxyl group of δ-tocopherol (**5**), and that the second α-d-glucopyranosyl residue and the inner β-d-glucopyranosyl residue, and the third α-d-glucopyranosyl residue and the second α-d-glucopyranosyl residue were 1,4-linked. Consequently, compound **8** was determined to be δ-tocopheryl β-malto-trioside.

Recently, the chemo-synthesis of α-tocopheryl β-glucoside and δ-tocopheryl β-glucoside by condensing each tocopherol with β-D-pentaacetylglucose followed by deacetylation [10] was reported. Compared to chemical glycosylation, that involves tedious procedures, including protection-deprotection of sugar hydroxyl groups, the present biocatalytic glycosylation by *K. pneumoniae* and CGTase is convenient for practical preparation of a series of tocopheryl β-maltooligosaccharides, the polymerization degrees of which are different by a glucose unit, at the same time.

### 2.2. Anti-allergic activity of β-glycosides of α- and δ-tocopherols

Recently, tocopherols have been reported to show anti-allergic activity such as anti-inflammatory effects [8,9]. The effects of α-tocopheryl β-glycosides **2**-**4** and δ-tocopheryl β-glycosides **6**-**8** on IgE antibody formation were investigated by an *in vivo* bioassay using glutenin as an antigen [17]. The average of rat plasma IgE level after treatment of glutenin with or without test compounds was examined and the results summarized in Table 1. The inhibitory activity of α-tocopheryl β-glucoside (**2**), δ-tocopheryl β-glucoside (**6**), and δ-tocopheryl β-maltoside (**7**)was much higher than that of the positive control, hydrocortisone. δ-Tocopheryl β-glucoside (**6**) showed the strongest suppressive action among the six tocopheryl β-glycosides. The inhibitory activity decreased in the order of δ-tocopheryl β-glucoside (**6**), α-tocopheryl β-glucoside (**2**), and δ-tocopheryl β-maltoside (**7**).

Next, the effects of tocopheryl β-glycosides **2**-**4** and **6**-**8** oncompound 48/80**-**induced histamine release [18] from rat peritoneal mast cells were examined. A high level of histamine (control, 41%) was released from rat peritoneal mast cells, which had been stimulated with 0.35 μg/mL of compound 48/80 (Table 2). α-Tocopheryl β-glucoside (**2**) effectively inhibited the compound 48/80**-**induced histamine release from rat peritoneal mast cells; %inhibition of **2** was 58%. The inhibitory activities of δ-tocopheryl β-glucoside (**6**) and δ-tocopheryl β-maltoside (**7**) were 63 and 41%, respectively. These results suggested that tocopheryl β-glycosides **2**, **6**, and **7** could be useful as anti-allergic agents. The anti-allergic activity such as inhibitory effects on IgE antibody formation and on histamine release of α- and δ-tocopherols **1** and **5** was higher than their glycosylated forms **2**-**4** and **6**-**8**. δ-Tocopheryl glucoside has been reported to act as potential tocopherol prodrug that is enzymatically hydrolyzed to release δ-tocopherol [19]. It is not clear whether the anti-allergic activity of tocopheryl glycosides synthesized here reflected that of tocopherols which had been released from their glycoside forms.

**Table 1:** Effects of tocopheryl β-glycosides **2**-**4** and **6**-**8** on IgE antibody formation.

Compound	IgE level^a^
None	384.0 ± 128.0
**1**	146.3 ± 44.8**
**2**	149.3 ± 47.7**
**3**	298.7 ± 95.4
**4**	320.0 ± 143.1
**5**	128.0 ± 64.0**
**6**	138.7 ± 57.4**
**7**	170.7 ± 60.3*
**8**	309.3 ± 158.6
Hydrocortisone	341.3 ± 120.6

^a^The results were expressed as average of plasma IgE level of six rats administered each test compound. Data are presented as mean ± SE. Asterisks indicate significant differences from controls as follows: *P<0.05 and **P<0.01.

**Table 2:** Effects of tocopheryl β-glycosides **2**-**4** and **6**-**8** on histamine release from rat peritoneal mast cells.

Compound	Histamine release (%)^a^
None	41
**1**	14
**2**	17
**3**	40
**4**	42
**5**	10
**6**	15
**7**	24
**8**	39

^a^Compound 48/80 (0.35 μg/mL)-induced histamine release from rat peritoneal mast cells after treatment with or without test sample.

Recently, glycosylation of physiologically active compounds has been reported to drastically improve their water-solubility [16]. Furthermore, it has been reported that α-tocopheryl β-glucoside and α-tocopheryl α-mannoside (tocopherol derivatives), which have been synthesized chemically, had excellent anti-allergic and anti-inflammatory activities, *i.e.,* these glycosides strongly inhibited histamine release from mast cells and suppressed IgE antibody formation [10,11]. The results obtained here demonstrate that sequential biocatalytic glycosylation by *K. pneumoniae* and CGTase is useful to prepare tocopheryl β-glycosides as water-soluble tocopherol derivatives. These tocopheryl β-glycosides would be food-additives which show potent anti-allergic activity. It has been reported that superoxide anion (O_2_^-^), which is generated primarily through the activation of the plasma membrane-bound NADPH-oxidase system from neutrophils, is responsible for allergic reactions [18,20]. Anti-allergic drugs such as epinastine and mequitazine have been reported to inhibit O_2_^-^ generation [18]. We reported that α-tocopheryl glucoside effectively inhibited O_2_^-^ generation from rat neutrophils [21]. Studies on the mechanism of tocopheryl glycosides synthesized here as anti-allergic compounds are now in progress.

## 3. Experimental

### 3.1. General

Substrates, α- and δ-tocopherols which were (*R,R,R*)-stereoisomers, were purchased from Sigma-Aldrich Co. CGTase was purchased from Amano Pharmaceutical Co. Ltd. The NMR spectra were recorded in CD_3_OD using a Varian XL-400 spectrometer. The chemical shifts were expressed in δ(ppm) referring to tetramethylsilane. The HRFABMS spectra were measured using a JEOL MStation JMS-700 spectrometer. HPLC was carried out on a CrestPak C18S column (150 × 4.6 mm) [solvent: MeOH:H_2_O (9:11, v/v); detection: UV (280 nm); flow rate: 1.0 mL/min]. 

### 3.2. Bacterial strain and culture conditions

*K. pneumoniae*, *X. campestris*, and *L. delbrueckii* were obtained from Sunny Health Co. Ltd., Japan. Culture medium used as a base for biotransformation experiments with *K. pneumoniae* had the following composition (in grams per liter): 4 g Bacto-peptone, 4 g yeast extract, and 25 g glycerol. The culture medium for *X. campestris* was composed of 10 g of maltose, 6 g of Bacto-peptone, 0.8 g of yeast extract, and 0.4 g of MgSO_4_, and that for *L. delbrueckii* consisted of 20 g of lactose, 5 g of yeast nitrogen base, 20 g of Bacto-casitone, 1 g of sorbitan monooleate, 2 g of K_2_HPO_4_, 0.1 g of MgSO_4_, 0.05 g of MnSO_4_, 2 g of ammonium citrate, and 5 g of sodium acetate. The cells were grown with continuous shaking on a rotary shaker (120 rpm) at 30 °C.

### 3.3. Bacterial glucosylation of tocopherols

Each culture of three bacteria was individually grown in 500-mL conical flasks containing 200 mL of culture medium at 30 °C. Prior to use for the experiments, the cells were harvested by centrifugation at 8,000 g for 15 min. Tocopheryl β-glucosides were prepared as follows. A total of 2 mmol of tocopherol (0.2 mmol/flask) was added to ten 300-mL conical flasks containing 5 g of bacterium cells and 1 g of glucose in 100 mL of freshly prepared culture medium. The mixture was incubated with continuous shaking on a rotary shaker (120 rpm) for 48 h at 30 °C. The reaction mixture was centrifuged at 8000 g for 15 min to remove the cells and the supernatant was extracted with *n*-butanol. The *n*-butanol fraction was purified by preparative HPLC to give tocopheryl β-glucoside.

### 3.4. Production of tocopheryl β-maltooligosaccharides by CGTase

To a solution containing 0.1 mmol of tocopheryl β-glucoside and 5 g of starch in 25 mM of sodium phosphate buffer (pH 7.0) was added 100 U of CGTase. After stirring of the reaction mixture at 40 °C for 24 h, the mixture was centrifuged at 3,000 g for 10 min. The supernatant was subjected on to a Sephadex G-25 column equilibrated with water to remove CGTase. The fractions containing glycosides were purified by preparative HPLC to give two tocopheryl β-glucooligosaccharides, *i.e.,* tocopheryl β-maltoside and tocopheryl β-maltotrioside. Spectral data of new compounds are as follows:

*δ-Tocopheryl β-maltoside* (**7**): White amorphous powder; FABMS *m/z*: 749.4455 [M+Na]^+^; ^1^H-NMR (400 MHz, CD_3_OD, δ in ppm): δ 0.86 (6H, d, *J* = 6.8 Hz, 13-CH_3_, 17-CH_3_), 0.87 (6H, d, *J* = 6.8 Hz, 21-CH_3_, H-22), 1.01-1.51 (18H, m, H-11, 12, 13, 14, 15, 16, 17, 18, 19, 20), 1.25 (3H, s, 1-CH_3_), 1.50-1.56 (3H, m, H-10, 21), 2.09 (3H, s, 8-CH_3_), 2.73 (2H, t, *J* = 6.2 Hz, H-3), 2.76 (2H, m, H-2), 3.09-3.99 (12H, m, H-2', 2'', 3', 3'', 4', 4'', 5', 5'', 6', 6''), 4.41 (1H, d, *J* = 7.6 Hz, H-1'), 5.11 (1H, d, *J* = 3.2 Hz, H-1''), 6.69 (1H, d, *J* = 2.8 Hz, H-5), 6.73 (1H, d, *J* = 2.8 Hz, H-7); ^13^C-NMR (100 MHz, CD_3_OD, δ in ppm): δ 16.3 (8-CH_3_), 20.4 (13-CH_3_, 17-CH_3_), 22.0 (C-11), 23.0 (21-CH_3_, C-22), 23.7 (C-3), 24.5 (1-CH_3_), 25.4 (C-15), 25.7 (C-19), 28.8 (C-21), 32.6 (C-2), 33.9 (C-13, C-17), 38.5 (C-12, C-14, C-16, C-18), 40.5 (C-20), 40.8 (C-10), 61.5 (C-6''), 62.8 (C-6'), 71.5 (C-4''), 74.1 (C-2''), 74.2 (C-5''), 74.8 (C-2'), 75.1 (C-3''), 76.7 (C-1), 76.8 (C-5'), 77.6 (C-3'), 80.7 (C-4'), 101.5 (C-1'), 102.9 (C-1''), 116.3 (C-5), 118.7 (C-7), 122.2 (C-4), 127.6 (C-8), 148.5 (C-9), 151.5 (C-6).

*δ-Tocopheryl β-maltotrioside* (**8**): White amorphous powder; FABMS *m/z*: 911.4851 [M+Na]^+^; ^1^H- NMR (CD_3_OD): δ 0.86 (6H, d, *J* = 6.8 Hz, 13-CH_3_, 17-CH_3_), 0.87 (6H, d, *J* = 6.8 Hz, 21-CH_3_, H-22), 1.00-1.55 (18H, m, H-11, 12, 13, 14, 15, 16, 17, 18, 19, 20), 1.25 (3H, s, 1-CH_3_), 1.51-1.57 (3H, m, H-10, 21), 2.09 (3H, s, 8-CH_3_), 2.73 (2H, t, *J* = 6.2 Hz, H-3), 2.77 (2H, m, H-2), 3.05-3.99 (18H, m, H-2', 2'', 2''', 3', 3'', 3''', 4', 4'', 4''', 5', 5'', 5''', 6', 6'', 6'''), 4.37 (1H, d, *J* = 8.0 Hz, H-1'), 4.59 (1H, d, *J* = 3.6 Hz, H-1''), 5.10 (1H, d, *J* = 3.2 Hz, H-1''') 6.69 (1H, d, *J* = 2.8 Hz, H-5), 6.73 (1H, d, *J* = 2.8 Hz, H-7); ^13^C- NMR (CD_3_OD): δ 16.3 (8-CH_3_), 20.5 (13-CH_3_, 17-CH_3_), 22.0 (C-11), 22.9 (21-CH_3_, C-22), 23.7 (C-3), 24.5 (1-CH_3_), 25.4 (C-15), 25.5 (C-19), 28.8 (C-21), 32.6 (C-2), 33.9 (C-13, C-17), 38.5 (C-12, C-14, C-16, C-18), 40.5 (C-20), 40.8 (C-10), 61.9 (C-6''), 62.2 (C-6'''), 62.5 (C-6'), 70.8 (C-4'''), 73.5 (C-5''), 73.7 (C-2''), 74.1 (C-2'''), 74.5 (C-3''', C-5'''), 75.1 (C-2', C-3''), 76.7 (C-1), 76.8 (C-5'), 77.7 (C-3'), 80.5 (C-4'), 81.5 (C-4''), 102.0 (C-1'), 102.5 (C-1''), 102.8 (C-1'''), 116.3 (C-5), 118.9 (C-7), 122.2 (C-4), 127.6 (C-8), 148.5 (C-9), 151.5 (C-6).

### 3.5. Suppressive action on IgE antibody formation

The inhibitory action of tocopheryl β-glycosides on IgE antibody formation was examined as follows. Glutenin (1 mg/rat) was used as the antigen, and Al(OH)_3_ and pertussis toxin were used as the adjuvants (20 mg and 0.6 mL/rat, respectively). Sensitization was made by injection of a mixture (0.6 mL) of the antigen and the adjuvant into the paws of each rat (male, ca. 200 g). Paw edema was measured 24 h after injection and the treated rats were divided in groups with an equal average swelling volume. Each sample was dissolved in physiological saline containing 10% Nikkol and the solution containing 0.4 mg of sample was injected daily into each rat for 11 d starting on the day of grouping. Hydrocortisone was used as the positive control. The amount of IgE was measured by the passive cutaneous anaphylaxis method on the 15th day [17]. The results were expressed as average of plasma IgE level of 6 rats administered each test compound.

### 3.6. Inhibitory action on histamine release from rat peritoneal mast cells

Effects of tocopheryl β-glycosides on histamine release from rat peritoneal mast cells were determned according to the previously reported method [18]. Peritoneal mast cells were collected from the abdominal cavity of rats (male Wistar rats, Nippon SLC) and purified to a level higher than 95%. The purified mast cells were suspended in a physiological buffered solution containing 145 mM NaCl, 2.7 mM KCl, 1.0 mM CaCl_2_, 5.6 mM glucose, and 20 mM HEPES (pH 7.4) to give approximately 10^4^ mast cells/mL. Cell viability was always greater than 90% as judged by the trypan blue exclusion test. Mast cells were preincubated with the test compound (1 μM) for 15 min at 37 °C, and subsequently exposed to compound 48/80 at 0.35 μg/mL. Histamine release was determined by a fluorometric assay according to the previously reported method [18], and was expressed as a percentage of total histamine.

## 4. Conclusions

Tocopheryl β-glycosides (tocopherol derivatives), *i.e.,* β-glucosides, β-maltosides, and β-maltotriosides of α- and δ-tocopherols, were successfully synthesized through sequential biocatalytic glycosylation by *K. pneumoniae* and CGTase. Three tocopheryl β-glycosides showed suppressive action on IgE antibody formation and exhibited inhibitory effects on histamine release from rat peritoneal mast cells. Particularly, the β-glucosides of both α- and δ-tocopherols showed significant anti-allergic activity among the tocopheryl β-glycosides synthesized here. It should be emphasized that the two biocatalysts, *i.e., K. pneumoniae* and CGTase, are potentially useful for practical production of tocopheryl glycosides as food additives.

## References

[1] Evans H.M., Bishop K.S. (1922). On the existence of a hitherto unrecognized dietary factor essential for reproduction. Science.

[2] Packer L. (1994). Vitamin E is nature’s master antioxidant. Sci. Am. Sci. Med..

[3] Kamal-Eldin A., Appelqvist L.A. (1996). The chemistry and antioxidant properties of tocopherols and tocotrienols. Lipids.

[4] Rimm E.R., Stampfer M.J., Ascherio A., Giovannucci E., Colditz G.A., Willett W.C. (1993). Vitamin E consumption and the risk of coronary heart disease in men. N. Engl. J. Med..

[5] Stampfer M., Hennekens C., Manson J., Colditz G., Rosner B., Willett W. (1993). Vitamin E consumption and the risk of coronary disease in women. N. Engl. J. Med..

[6] Brigelius-Flohe R., Traber M.G. (1999). Vitamin E: Function and metabolism. FASEB J..

[7] Engin K.N. (2009). Alpha-tocopherol: looking beyond an antioxidant. Mol. Vis..

[8] Ju J., Hao X., Lee M.J., Lambert J.D., Lu G., Xiao H., Newmark H.L., Yang C.S. (2009). A gamma-tocopherol-rich mixture of tocopherols inhibits colon inflammation and carcinogenesis in azoxymethane and dextran sulfate sodium-treated mice. Cancer Prev. Res..

[9] Rizzo M.R., Abbatecola A.M., Barbieri M., Vietri M.T., Cioffi M., Grella R., Molinari A., Forsey R., Powell J., Paolisso G. (2008). Evidence for anti-inflammatory effects of combined administration of vitamin E and C in older persons with impaired fasting glucose: Impact on insulin action. J. Am. Coll. Nutr..

[10] Satoh T., Miyataka H., Yamamoto K., Hirano T. (2001). Synthesis and physiological activity of novel tocopheryl glycosides. Chem. Pharm. Bull..

[11] Uhrig R.K., Picard M.A., Beyreuther K., Wiessler M. (2000). Synthesis of antioxidative and anti-inflammatory drugs glucoconjugates. Carbohydr. Res..

[12] Sanford P.A. (1979). Exocellular microbial polysaccharides. Adv. Carbohydr. Chem. Biochem..

[13] Welman A.D., Maddox I.S. (2003). Fermentation performance of an exopolysaccharide-producing strain of *Lactobacillus delbrueckii* subsp. *bulgaricus*. J. Ind. Microbiol. Biotechnol..

[14] Faria S., Vieira P.A., Resende M.M., Franca F.P., Cardose V.L. (2009). A comparison between shaker and bioreactor performance based on the kinetic parameters of xanthan gum production. Appl. Biochem. Biotechnol..

[15] Lahmann M., Thiem J. (1997). Synthesis of α-tocopheryl oligosaccharides. Carbohydr. Res..

[16] Shimoda K., Hamada H., Hamada H. (2008). Chemo-enzymatic synthesis of ester-linked taxol-oligosaccharide conjugates as potential prodrugs. Tetrahedron Lett..

[17] Koda A., Miura T., Inagaki N., Sakamoto O., Arimura A., Nagai H., Mori H. (1990). A method for evaluating anti-allergic drugs by simultaneously induced passive cutaneous anaphylaxis and mediator cutaneous reactions. Int. Arch. Allergy. Appl. Immunol..

[18] Akagi M., Katakuse Y., Fukuishi N., Kan T., Akagi R. (1994). Superoxide anion-induced histamine release from rat peritoneal mast cells. Biol. Pharm. Bull..

[19] Mavon A., Raufast V., Redoules D. (2004). Skin absorption and metabolism of a new vitamin E prodrug, δ-tocopherol-glucoside: *In vitro* evaluation in human skin models. J. Controll. Rel..

[20] Abo A., Boyhan A., West I., Thrasher A.G., Segal A.W. (1992). Reconstitution of neutrophil NADPH oxidase activity in the cell-free system by four components: p67-phox, p47-phox, p21rac1, and cytochrome b-245. J. Biol. Chem..

[21] Shimoda K., Kondo Y., Akagi M., Abe K., Hamada H., Hamada H. (2007). Synthesis of α-tocopheryl disaccharides as potential antiallergic agents. Chem. Lett..

